# Atmospheric cold plasma treatment effects on quality of cloudy apple juice during storage

**DOI:** 10.1111/1750-3841.70158

**Published:** 2025-03-26

**Authors:** Emine Ozen, Abhinav Mishra, Rakesh K. Singh

**Affiliations:** ^1^ Department of Food Science and Technology University of Georgia Athens Georgia USA; ^2^ Vocational School of Technical Sciences Food Processing Department Ardahan University Ardahan Turkey

**Keywords:** apple juice, atmospheric cold plasma, beverage, nonthermal processing, shelf life

## Abstract

Atmospheric cold plasma (ACP) is a nonthermal technology that shows promise for use in food processing. This study evaluated the effects of ACP treatment on the quality of cloudy apple juice using two different feed gases—simulated air (SA), consisting of 80% nitrogen and 20% oxygen, and combined gas (CG), made up of 90% nitrogen and 10% oxygen—for varying durations (30–150 s). The impact of storage at 4°C for 3 weeks on physicochemical properties (pH, color, viscosity, titratable acidity, soluble solids) and bioactive compounds (total phenolic content [TPC], antioxidant capacity) was assessed. Microbial survival, including total plate count, yeast and mold counts, and *Alicyclobacillus acidoterrestris* spores, was also evaluated. ACP treatment did not significantly alter pH, °Brix, or viscosity immediately post‐treatment; however, pH decreased significantly after storage (e.g., SA at 30 s: 3.66 ± 0.0; CG at 120 s: 3.65 ± 0.01). Post‐storage, ACP‐treated juices exhibited reduced lightness (*L*) and increased chroma (*C*), particularly at longer treatment times (150 s). TPC initially decreased with prolonged ACP exposure but increased post‐storage, suggesting plasma‐induced cell wall disruption facilitated phenolic release. Antioxidant activity remained stable in ACP‐treated juices, in contrast to thermally pasteurized juices, which showed higher DPPH inhibition. ACP achieved limited reduction of *A. acidoterrestris* spores (1.06 ± 0.35 log CFU/mL with SA for 3 min) and had no significant effect on yeast/mold counts, which increased during storage. No bacterial growth was detected in ACP‐treated juices, likely due to the acidic environment.

## 1 INTRODUCTION

Fruit juices are a valuable source of essential nutrients and bioactive compounds that cater to consumer preferences for health, taste, and convenience (Carrillo et al., 2014; Gomes et al., 2017). Cloudy apple juice (CAJ) is particularly beneficial due to its higher content of prebiotics and bioactive compounds, such as pectin, polyphenols, and ascorbic acid compared to clear apple juice (Fonteles & Rodrigues, 2018; Oszmianski et al., 2007). To ensure safety and prolong shelf life, juices are conventionally pasteurized through thermal processing, which effectively inactivates spoilage microorganisms and enzymes (Gonzalez & Barrett, 2010). However, thermal treatment can significantly alter sensory and quality parameters, including color, vitamin content, and phenolic composition (Gonzalez & Barrett, 2010; Wang et al., 2012). The juice industry faces an ongoing challenge in maintaining fresh‐like quality while extending shelf life (Ramos et al., 2013), prompting the exploration of nonthermal processing alternatives, such as pulsed electric fields (Buckow et al., 2013), ultrasound (Adekunte et al., 2010), pulsed light (Tremarin et al., 2017), high‐pressure processing (Marszałek et al., 2017), and membrane filtration (Gurak et al., 2010).

Atmospheric cold plasma (ACP) has emerged as an attractive nonthermal processing technology for the food industry (Ozen & Singh, 2020; Pankaj et al., 2014). ACP is a neutral, ionized gas composed of electrons, free radicals, and reactive species at near‐room temperature (Niemira, 2012). Depending on the feed gas composition, ACP generates reactive oxygen and nitrogen species, such as singlet oxygen, hydroxyl (OH), and nitric oxide (NO) radicals, which influence microbial inactivation and food quality (Pankaj et al., 2018). The effectiveness of ACP depends on plasma source parameters, including feed gas type, gas flow rate, and applied voltage (Pankaj et al., 2014; Ramos et al., 2013).

Nonthermal processing of fruit juices using ACP represents a promising area of research due to its potential impact on juice quality and safety. ACP has been applied to a variety of juices, including apples (Dasan & Boyaci, 2018; Farias et al., 2020; Illera et al., 2019; Liao et al., 2018; Xiang et al., 2018), pineapple (Pipliya et al., 2022), carrot (Umair et al., 2019), and orange juices (Xu et al., 2017). However, limited research has explored ACP treatment of CAJ and its quality changes during storage. Notably, Ozen et al. (2022) processed apple juice using the same ACP device and parameters as this study, achieving a 5‐log CFU/mL reduction in *Escherichia coli* O157:H7 within 90 s, confirming the microbiological safety of the process.

Although ACP has been investigated in various liquid foods, this study presents a novel contribution by focusing on CAJ, which poses unique challenges due to its high turbidity and complex composition (Ozen et al., 2022; Pankaj et al., 2018). Compared to clear juices, CAJ has received less attention in ACP research (Farias et al., 2020; Illera et al., 2019), making this study a valuable addition to existing literature. Additionally, this study examines the effects of different feed gases and storage conditions on plasma‐treated CAJ, an area previously underexplored. Research on ACP's long‐term effects on juice shelf life and quality, particularly bioactive compound retention, remains scarce, and this study aims to address these gaps (Ozen et al., 2022; Pankaj et al., 2018). Furthermore, microbial inactivation achieved through ACP requires optimization, and this study highlights future research directions for enhancing food safety (Ozen & Singh, 2020; Pankaj et al., 2014).

The objective of this study was to evaluate the effects of ACP processing on CAJ quality, including color, antioxidant activity, and turbidity. A comparative analysis was conducted among ACP‐treated, non‐processed, and thermally processed CAJ. Additionally, two distinct gas mixtures were used as ACP feed gases—simulated air (SA) (80% nitrogen, 20% oxygen) and combined gas (CG) (90% nitrogen, 10% oxygen)—to assess the influence of gas composition on juice quality over varying treatment durations and 3 weeks of storage.

## 2 MATERIALS AND METHODS

### 2.1 Sample preparation

The study utilized Red Delicious apples obtained locally from a market in Georgia, USA, and they were kept at 4°C for one night. The apples underwent cleaning through brushing and washing to eliminate surface contaminants and wax coating. Following cleaning, the apples were halved and processed using a screw extractor (A200, Hobart Co.). The extracted juice was strained through triple‐layered cheesecloth. Ascorbic acid (0.1%) was added to prevent enzymatic browning reactions. Juice preparation was conducted in a temperature‐controlled environment at 4°C, and samples were stored at −18°C until needed. For studying the ACP‐induced inactivation of *Alicyclobacillus acidoterrestris*, commercially pasteurized clear apple juice (Great Value Ltd.) was used. The CAJ was selected to eliminate particulates that could shield *A. acidoterrestris* spores from ACP treatment, ensuring a uniform medium and accurate inactivation results. Additionally, pasteurized apple juice was used to remove background flora, preventing interference from other microorganisms and ensuring that the observed effects were solely attributable to the ACP treatment on *A. acidoterrestris* spores.

### 2.2 ACP treatments

The ACP device utilizing a jet based dielectric barrier discharge technology (CD50, Plasmatreat) used in this study to process CAJs is illustrated and described in Sharma and Singh (2022) and depicted in Ozen et al. (2024). The system uses pulsed DC voltage to generate plasma. The device components include 1 kV‐16 A power generator (FG5001), a plasma jet (CD50), and a high‐voltage transformer (HTR11). A transformer stepped up the output voltage from 1 to 20 kV for ignition and 2 kV for the arc drop voltage, operating within a frequency range of 15–25 Hz. During the experiment, a blend of oxygen and nitrogen gases was used as the feed to flow through the electrodes, producing a plasma jet rich in active species. The gas cylinder pressure was reduced via regulators to enter the plasma device at approximately 13 kPa gage pressure and exit the nozzle as plasma slightly above the atmospheric pressure. Each gas was passed through a separate rotameter (Cole‐Parmer) for direct measurement and control of the flow rate. To direct the ACP into CAJ, a polytetrafluoroethylene (PTFE) tube (3 mm inner diameter, 5 mm outer diameter, and 12.5 cm in length) was attached to the nozzle. As a result of the plasma passing through the CAJ, bubbles were generated inside the sample which caused mixing of the liquid. The gas mixtures used in this study were as follows: (i) SA comprised of 80% nitrogen and 20% oxygen to mimic the composition of atmospheric air and (ii) combined air (CG) composed of 90% nitrogen and 10% oxygen. The processing times were 30, 60, 90, 120, and 150 s. A 1‐L flask filled with 100 mL of CAJ was positioned at 28.5 cm from the plasma nozzle's outlet under the plasma nozzle. To ensure good mixing and direct contact with the plasma species, PTFE was placed into the sample at a depth of 0.7 cm, and the plasma flow rates were 5 L/min.

Inactivation of *A. acidoterrestris* spores by ACP was carried out using 20 mL pasteurized CAJ to eliminate background flora that could interfere with the experiments and ensure that the observed effects on spore inactivation were solely attributable to the *A. acidoterrestris* spores treated by ACP. A 50 mL Falcon^®^ centrifuge tube was filled with 20 mL of *A. acidoterrestris* spores inoculated juice and was positioned at 12.5 cm from the plasma nozzle's outlet under the plasma nozzle. Nozzles were placed inside the juice and were positioned so that they were 2.5 cm deep from the surface of the juice.

### 2.3 Batch pasteurization

The thermal processing was performed using batch treatment in a water bath (SHWB10, Cole‐Parmer Instrument Co.). For each sample, 200 mL of CAJ was placed in a 250 mL wide‐mouth polypropylene container (Fisher Scientific). Temperature monitoring was achieved by connecting thermocouple wires through the bottle caps to a data logger (Fluke 52II, FLUKE). Based on parameters established by Renard and Maingonnat (2012), the juice was heated at 90°C for 1 min. Following the heat treatment, samples were rapidly cooled in an ice bath and subsequently refrigerated at 4°C for a 3‐week period.

### 2.4 Color, pH, °brix, titratable acidity, and reducing sugar measurements

The CIELAB coordinates of the juice samples were measured with a colorimeter (EZ 4500 L, Hunter Associates Laboratory Inc.). Before measurement, white and black tiles were used for calibration of the colorimeter. For protection against the possible effects of surrounding light, 35 mL apple juice was placed in an opaque foam box. The *L* ^∗^ (lightness), *a* ^∗^ (greenness/redness), and *b* ^∗^ (blueness/yellowness) values of the juice samples were measured. The following equations were used to calculate the chroma and hue angle in each juice sample:

(1) $$C^{*}={\left(a^{*2}+b^{*2}\right)}^{\frac{1}{2}}$$

(2) $$h=\mathrm{archtan}\left(\frac{b^{*}}{a^{*}}\right)$$

The pH and soluble solid content of the samples were analyzed with a pH meter (Accumet AB150, Fisherbrand, OTT) and a refractometer (MA 884, Milwaukee Instruments Inc.). Titratable acidity (TA) in apple juices was determined according to the AOAC (2016) method no. 942.15 and reported as grams of malic acid per 100 mL of juice. The method of Miller (1959) 3,5‐dinitrosalicylic acid was applied to measure the reducing sugars.

### 2.5 Turbidity, cloud stability, and particle size distribution (PSD) measurements

Turbidity or cloud value of CAJ was determined following the method of Bhat and Goh (2017). A spectrometer (Model #1200, Cole‐Parmer Instrument Co.) was used to measure the absorbance of the samples at 660 nm, and turbidity was calculated using Equations (3) and (4).

Cloud stability of CAJ was assessed and expressed as relative turbidity (%*T*), based on the procedure by de Paepe et al. (2015). The absorbance at 660 nm was initially measured for the samples (*T*_0_). After centrifugation at 4200 × *g* for 10 min, the absorbance of the supernatant was measured (*T_c_*). Cloud stability was then calculated according to Equation (5):

(3) $$\mathrm{Transmittance}=100\times 10^{-\mathrm{Absorbance}}$$

(4) $$\mathrm{Turbidity}\;\left(T\right)=100-\mathrm{Tranmittance}$$

(5) $$\%T=\frac{T_{c}}{T_{0}}\;\times 100$$

The particle size distribution (PSD) of the samples was analyzed using a particle size analyzer (MAM 5004, Malvern Instrument Ltd.). Laser light diffraction was used to measure particles ranging from 0.1 to 1000 µm. The software of the analyzer calculated the volume mean diameter (*D*[4,3]), the surface area mean diameter (*D*[3,2]), as well as the values for *d*(0.1), *d*(0.5), and *d*(0.9), which represent the particle sizes below which 10%, 50%, and 90% of the total particles, respectively, are found.

### 2.6 Total phenolic content (TPC) and antioxidant activity determination

Total phenolic content (TPC) was measured using the Folin–Ciocalteu method described by Singleton et al. (1999). Two hundred microliters (µL) of juice samples were diluted with 1.8 mL of distilled water. A combination of 3 mL water, 200 µL Folin–Ciocalteu reagent (Sigma‐Aldrich Ltd.,), and 200 µL diluted juice was mixed and waited for 10 min. Thereafter, 600 µL of sodium carbonate solution (80 µL, 20 g/dL) was added. The mixture was incubated at 30°C for 2 h. Absorbance of the samples was determined at 762 nm. Gallic acid solutions were utilized to generate a standard curve, and the results were reported as milligrams of gallic acid equivalent per liter of apple juice (mg GAE/L).

Antioxidant capacity was determined by the DPPH assay described by Illera et al. (2019).

The sample was mixed with equal volume of ethanol and centrifuged at 5000 × *g* for 5 min. Next, 3.5 mL of 0.2 mM DPPH (Cayman Chemical Inc) was added to 1 mL of the supernatant. The mixture was incubated at room temperature (22°C) for 30 min, and the absorbance of the sample was measured at 517 nm. A control solution was prepared using 1 mL of ethanol in the DPPH solution. The antioxidant inhibition was calculated by the following equation:

(6) $$\mathrm{The}\;\mathrm{DPPH}\;\mathrm{inhibition}\;\left(\%\right)=\left(1-\frac{A_{s}}{A_{c}}\right)\;\times 100$$

where *A_s_* and *A_c_* indicate the absorbance of the sample and control, respectively.

### 2.7 Microbial quality measurements

Plate count agar (PCA; Difco Ltd., BD) and Dichloran Rose Bengal Chloramphenicol (DRBC; Difco Ltd., BD) were used to enumerating the total plate count and total fungal count, respectively, using 0.1 mL for plating. The PCA plates were kept in an incubator at 37°C for 24 h, whereas the DRBC plates were incubated at 25°C for 5 days. The results are presented as the number of colony‐forming units (CFU)/mL.

To inactivate *A. acidoterrestris* (ATCC 49025), orange serum agar was adjusted to pH 3.7 with 10% tartaric acid, cultured for 2 days at 43°C, and subcultured monthly at 4°C. After streaking the growth cultures onto potato dextrose agar adjusted to pH 5.6 with 10% tartaric acid, they were incubated at 43°C for 7 days. Then, spores were stained by the Schaeffer–Fulton method described by Schaeffer (1933), the sporulation was checked by microscopy (expected: at least 80% sporulation). Afterward, the sporulated cells were harvested and centrifuged for 20 min at 3000 × *g*. Pelleted cells were then inoculated into 20 mL of pasteurized apple juice. A final concentration of 10^5^–10^6^ spores/mL was achieved.

### 2.8 Statistical analysis

Each measurement was performed three times. To perform the statistical analysis, SPSS software version 20 was used (SPSS Inc.), where an analysis of variance was applied to evaluate the data. Statistical significance was established at *p* < 0.05.

## 3 RESULTS AND DISCUSSION

### 3.1 Effect of atmospheric cold plasma on physical attributes of CAJ

Tables 1 and 2 show the major physical characteristics of CAJ with thermal batch pasteurization, ACP processing, and no processing with and without shelf life, respectively. Acidity and pH are closely related to the quality of processed foods. Any drastic change in them could adversely affect consumer acceptability and shelf life of the foods. It was found that there was no significant difference in pH between samples that were thermally processed, ACP‐treated and samples that were not treated (*p* > 0.05) when pH of the samples were measured just after the treatment. Dasan and Boyaci (2018) and Tarabová et al. (2021) also found that ACP had no significant impact on apple juice pH. As cold plasma processing generally causes pH changes in juices, it is also predicted that the juice composition would act as a buffer to preserve pH (Lukes et al., 2014; Machala et al., 2019). However, compared to juices without processing, all CAJ processed with ACP had significantly lower pH after 3 weeks of storage (*p* < 0.05) (Table 2). The pH of juices significantly decreased after 30 s for SA from 3.8 ± 0.0 to 3.66 ± 0.0 and 120 s for CG from 3.8 ± 0.0 to 3.65 ± 0.01. Cold plasma discharge might affect juice acidity by solubilizing hydroxyl radicals (Pankaj et al., 2017), and pH might be affected by nitric and nitrous acids and hydrogen peroxide (H_2_O_2_) (Helmke et al., 2011; Liu et al., 2010; Oehmigen et al., 2010). Ozen et al. (2022) conducted optical absorption spectroscopy (OAS) on the device utilized in this study to examine the plasma composition of SA and CG following plasma exposure. The spectrum ranging from 190 to 308 nm exhibited ozone absorption (200–300 nm) along with peaks for NO and NO2 in the 190–225 nm and 230–280 nm ranges, respectively. These findings suggest that cold plasma treatment can generate reactive species, influencing the chemical properties and acidity of the juice.

**TABLE 1:** The effect of atmospheric cold plasma with simulated air (SA; 80% nitrogen and 20% oxygen) and combined gas (CG; 90% nitrogen and 10% oxygen) as working gasses for 30, 60, 90, 120, and 150 s on the quality of apple juice after the processing (without shelf life).

Treatment	°Brix	pH	Viscosity (10^−3^ Pa. s)	Color (*L*)	Color (Chroma)	Color (Hue)	TA (% malic acid)
SA30	12.80 ± 0.00^a^	3.8 ± 0.0^a^ ^b^	1.87 ± 0.01^a^	41.67 ± 6.32^a^ ^b^	49.85 ± 5.85^a^ ^b^	71.00 ± 4.96^a^	0.26 ± 0.04^a^
SA60	12.80 ± 0.28^a^	3.9 ± 0.0^b^	1.98 ± 0.04^a^	41.50 ± 5.38^a^ ^b^	51.30 ± 4.92^a^ ^b^	69.15 ± 3.96^a^	0.22 ± 0.03^a^
SA90	12.95 ± 0.35^a^	3.9 ± 0.1^a^ ^b^	1.99 ± 0.09^a^	41.06 ± 5.91^a^ ^b^	52.14 ± 3.95^b^	68.96 ± 4.49^a^	0.23 ± 0.00^a^
SA120	12.80 ± 0.14^a^	3.8 ± 0.0^a^ ^b^	1.92 ± 0.06^a^	24.32 ± 2.76^a^	32.58 ± 3.35^a^	60.29 ± 1.25^a^	0.27 ± 0.02^a^
SA150	13.25 ± 0.07^a^	3.8 ± 0.1^a^ ^b^	1.97 ± 0.17^a^	23.82 ± 2.59^a^	32.19 ± 4.08^a^	59.54 ± 1.05^a^	0.24 ± 0.04^a^
CG30	12.85 ± 0.07^a^	3.9 ± 0.1^a^ ^b^	2.04 ± 0.01^a^	36.72 ± 12.08^a^ ^b^	44.95 ± 11.29^a^ ^b^	67.39 ± 8.47^a^	0.25 ± 0.02^a^
CG60	12.65 ± 0.21^a^	3.8 ± 0.0^a^ ^b^	2.06 ± 0.06^a^	29.52 ± 2.55^a^ ^b^	39.86 ± 3.12^a^ ^b^	62.27 ± 1.99^a^	0.24 ± 0.02^a^
CG90	12.75 ± 0.35^a^	3.8 ± 0.0^a^ ^b^	2.05 ± 0.01^a^	25.82 ± 0.93^a^ ^b^	35.66 ± 1.32^a^ ^b^	59.63 ± 0.57^a^	0.24 ± 0.02^a^
CG120	12.15 ± 0.92^a^	3.8 ± 0.0^a^ ^b^	2.10 ± 0.33^a^	23.90 ± 1.71^a^	33.05 ± 1.81^a^ ^b^	58.72 ± 0.22^a^	0.23 ± 0.02^a^
CG150	12.85 ± 0.21^a^	3.7 ± 0.0^a^	2.04 ± 0.11^a^	31.07 ± 7.42^a^ ^b^	39.04 ± 4.89^a^ ^b^	64.97 ± 6.49^a^	0.25 ± 0.01^a^
Nontreated	12.50 ± 0.14^a^	3.8 ± 0.0^a^ ^b^	1.76 ± 0.11^a^	40.83 ± 9.71^a^ ^b^	47.78 ± 4.62^a^ ^b^	70.96 ± 8.45^a^	0.34 ± 0.09^a^
Pasteurized	13.00 ± 0.14^a^	3.8 ± 0.1^a^ ^b^	1.94 ± 0.18^a^	48.28 ± 1.21^b^	42.72 ± 0.14^a^ ^b^	74.06 ± 1.54^a^	0.24 ± 0.02^a^

*Note*: ^a^ and ^b^ mean in the same column followed by different superscript letters are significantly different (*p* < 0.05).

Abbreviation: TA, titratable acidity.

**TABLE 2:** The effect of atmospheric cold plasma with simulated air (SA; 80% nitrogen and 20% oxygen) and combined gas (CG; 90% nitrogen and 10% oxygen) as feed gasses for 30, 60, 90, 120, and 150 s on the quality of apple juice after 3 weeks storage at 4°C.

Treatment	°Brix	pH	Viscosity (10^−3^ Pa. s)	Color (*L*)	Color (Chroma)	Color (Hue)	TA (% malic acid)
SA30	11.80 ± 0.28^a^	3.66 ± 0.04^bc^	1.74 ± 0.02^a^	27.43 ± 1.39^bcd^	33.96 ± 1.79^b^	63.07 ± 0.76^a^ ^b^	0.26 ± 0.00^a^
SA60	11.70 ± 0.28^a^	3.61 ± 0.04^c^	1.75 ± 0.06^a^	28.42 ± 0.85^bcd^	34.57 ± 0.91^bd^	64.04 ± 0.06^a^	0.25 ± 0.02^a^
SA90	12.65 ± 0.49^a^	3.63 ± 0.04^c^	1.73 ± 0.06^a^	29.81 ± 0.87^bcd^	38.23 ± 0.65^a^ ^b^	63.96 ± 0.40^a^	0.26 ± 0.00^a^
SA120	11.10 ± 1.13^a^	3.62 ± 0.06^c^	1.72 ± 0.07^a^	31.17 ± 1.39^bd^	38.34 ± 1.87^a^ ^b^	64.76 ± 0.26^a^	0.23 ± 0.02^a^
SA150	11.70 ± 0.14^a^	3.62 ± 0.05^c^	1.69 ± 0.01^a^	32.03 ± 0.09^d^	39.17 ± 0.51^a^ ^b^	64.37 ± 0.69^a^	0.22 ± 0.07^a^
CG30	13.15 ± 0.07^a^	3.72 ± 0.01^a^ ^c^	1.85 ± 0.02^a^	25.10 ± 0.23^c^	33.50 ± 0.37^b^	60.73 ± 0.05^b^	0.24 ± 0.03^a^
CG60	12.60 ± 0.14^a^	3.69 ± 0.00^a^ ^c^	1.74 ± 0.05^a^	27.90 ± 0.16^bcd^	37.47 ± 0.84^a^ ^b^	62.44 ± 0.13^b^	0.23 ± 0.02^a^
CG90	12.40 ± 1.13^a^	3.67 ± 0.00^bc^	1.80 ± 0.10^a^	30.07 ± 0.25^bcd^	39.63 ± 0.46^a^ ^d^	64.35 ± 0.08^a^	0.24 ± 0.02^a^
CG120	12.60 ± 0.57^a^	3.65 ± 0.01^bc^	1.80 ± 0.12^a^	31.39 ± 0.01^d^	40.89 ± 0.28^a^	65.03 ± 0.06^a^	0.23 ± 0.00^a^
CG150	10.70 ± 0.85^a^	3.60 ± 0.01^c^	1.72 ± 0.02^a^	32.06 ± 0.83^d^	42.25 ± 1.64^a^ ^c^	65.43 ± 0.57^a^	0.23 ± 0.02^a^
Nontreated	11.60 ± 0.71^a^	3.81 ± 0.01^a^	1.70 ± 0.04^a^	26.30 ± 3.56^bc^	33.84 ± 3.95^bd^	62.51 ± 2.23^a^ ^b^	0.24 ± 0.02^a^
Pasteurized	12.10 ± 0.14^a^	3.76 ± 0.03^a^ ^b^	1.81 ± 0.10^a^	45.51 ± 0.27^a^	40.56 ± 0.01^a^	73.62 ± 0.11^c^	0.26 ± 0.00^a^

*Note*: ^a^, ^b^, ^c^, and ^d^ mean in the same column followed by different superscript letters are significantly different (*p* < 0.05).

Abbreviation: TA, titratable acidity.

Furthermore, these findings agree with those of Liao et al. (2018) and Xiang et al. (2018), finding that apple juice pH decreased after ACP processing. Changes in pH after cold plasma treatment are associated with juice type, plasma type, juice amount, plasma active species production, and so forth (Tarabová et al., 2021). The effects of treatment time and the types of gas used in the ACP processing on the pH of CAJ before and after storage were evaluated. The results indicated no significant effect of either processing time or gas type on the pH (*p* > 0.05). No significant change in TA was measured after treatment and after storage for 3 weeks (Table 2).

Sweetness in fruit juices is one of the important elements defined by °Brix degree, which indicates the percentage of water‐soluble solids in juices. There was no significant difference in °Brix among thermally processed, ACP processed, and non‐processed samples with and without storage (*p* > 0.05) (Tables 1 and 2). Although carbohydrates account for most soluble solids in juices, it is difficult to react to plasma‐generated species with these macromolecules due to the short lifetimes of the species (Liao et al., 2018). Viscosity, as a key physical property, often influences the quality of liquid food products (Saravacos, 1970). No significant difference in viscosity was observed between ACP treated and control samples before and after storage (*p* > 0.05) (Tables 1 and 2). It was also found that the processing time and gas type were not significantly (*p* > 0.05) had effect on °Brix and viscosity of all treated samples.

The color parameters for processed and non‐processed juice samples are given in Table 1 for samples without storage and Table 2 for samples stored for 3 weeks. The lightness (L*) represents the colors of white, gray, and black (achromatic colors) and its value range from 0 (black) to 100 (white). The *L*‐values of ACP‐treated samples did not show a significant difference compared to the nontreated samples (*p* > 0.05), both with and without storage (Tables 1 and 2). ACP‐processed juices with SA for 120 and 150 s and CG for 120 s showed significantly lower *L*‐values than thermally processed juices without storage. After 3 weeks of storage for ACP treatment, the *L*‐values ranged from 27.43 ± 1.39 to 32.03 ± 0.09 for SA and from 25.10 ± 0.23 to 32.06 ± 0.83 for CG (Table 2). *L*‐Values of ACP treated samples showed significantly lower values compared to thermally processed ones. Moreover, both gas compositions showed slightly increased *L*‐values with increased treatment time, but the change was insignificant. When the time reached 150 s for both gas mixtures, the CAJ color became significantly darker compared to unprocessed samples. ACP‐processed samples were all lighter than thermally processed samples. Moreover, *L*‐values were not significantly affected by the gas composition or processing time. The results agree with Liao et al. (2018), who found that increasing the plasma processing time decreased the lightness of apple juice. The polymerization of phenolic content and the oxidation of pigment compounds by cold plasma species might impact color change (Kovačević et al., 2016; Wang et al., 2012).

Chroma (C*) describes how vibrant or dull a color is, depending on whether it is pure or gray. As C* values increase, the color becomes more vivid. There was no significant difference between the ACP‐processed samples and the raw or thermally pasteurized samples without storage (Table 1). After 3 weeks of storage, the C*‐value of nontreated samples, 33.84 ± 3.95, was not significantly different from ACP‐treated samples, but the samples treated with CG for 120 and 150 s were higher than nontreated samples (Table 2). The lower treatment time for ACP treatment shows significantly lower C*‐values than pasteurized samples. It was observed that the C*‐values of ACP‐treated CAJ were decreased by increasing processing time when the values were measured just after treatment. The finding agreed with Dasan and Boyaci (2018) and Wang et al. (2023) finding that apple juice saturation increased with plasma processing time. On the other hand, the C*‐values of ACP‐treated samples increased with increasing treatment time after 3 weeks of storage, but the increase was not significant. The effects of the ACP processing time on C*‐values were not significant before and after storage compared to un‐treated and thermally processed samples, whereas C*‐values of ACP processed samples with CG showed significantly increasing with time. There was no significant effect of gas type measured in all ACP treated samples.

The Hue (*h*‐value) represents the visual perception of sample colors (red, yellow, green, and blue, or the combination of them). There was no significant difference in *h*‐values between the treatments when the measurement was completed just after the treatment (Table 1). Although the *h*‐values of pasteurized juices were not significantly different from ACP‐treated samples, pasteurized samples measured significantly higher after 3 weeks of storage (Table 2). Moreover, the gas mixture used in the plasma processing had no significant effect on *h*‐values.

**FIGURE 1:**
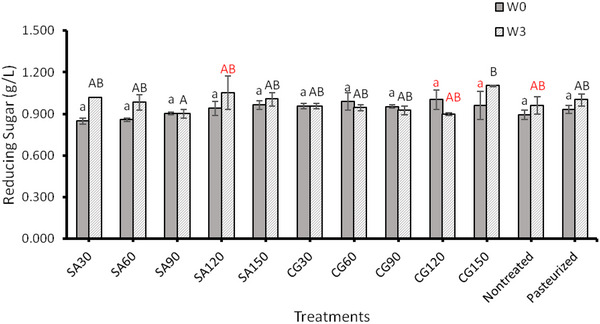
The effect of atmospheric cold plasma with simulated air (SA) (80% nitrogen and 20% oxygen) and combined gas (CG) (90% nitrogen and 10% oxygen) as feed gasses for 30, 60, 90, 120, and 150 s processing times on reducing sugar in apple juice after the processing (W0) and after 3 weeks of storage at 4°C (W3). Values followed by different superscript letters are significantly different (*p* < 0.05).

ACP treatment did not result in any significant changes in reducing sugar in either stored or non‐stored samples (*p* > 0.05) (Figure 1). The reducing sugar content of ACP‐treated CAJ is not affected by the gas composition used to produce plasma or by processing time except for CG after 150 s with 3 weeks of storage. This increase may be explained by the ozone generated by the ACP breaking the glycosidic bonds in the polysaccharides and degrading them into monosaccharides (Ben'Ko et al., 2013; Umair et al., 2019).

### 3.2 Effect of ACP on cloud value, cloud stability, and PSD of apple juice

**FIGURE 2:**
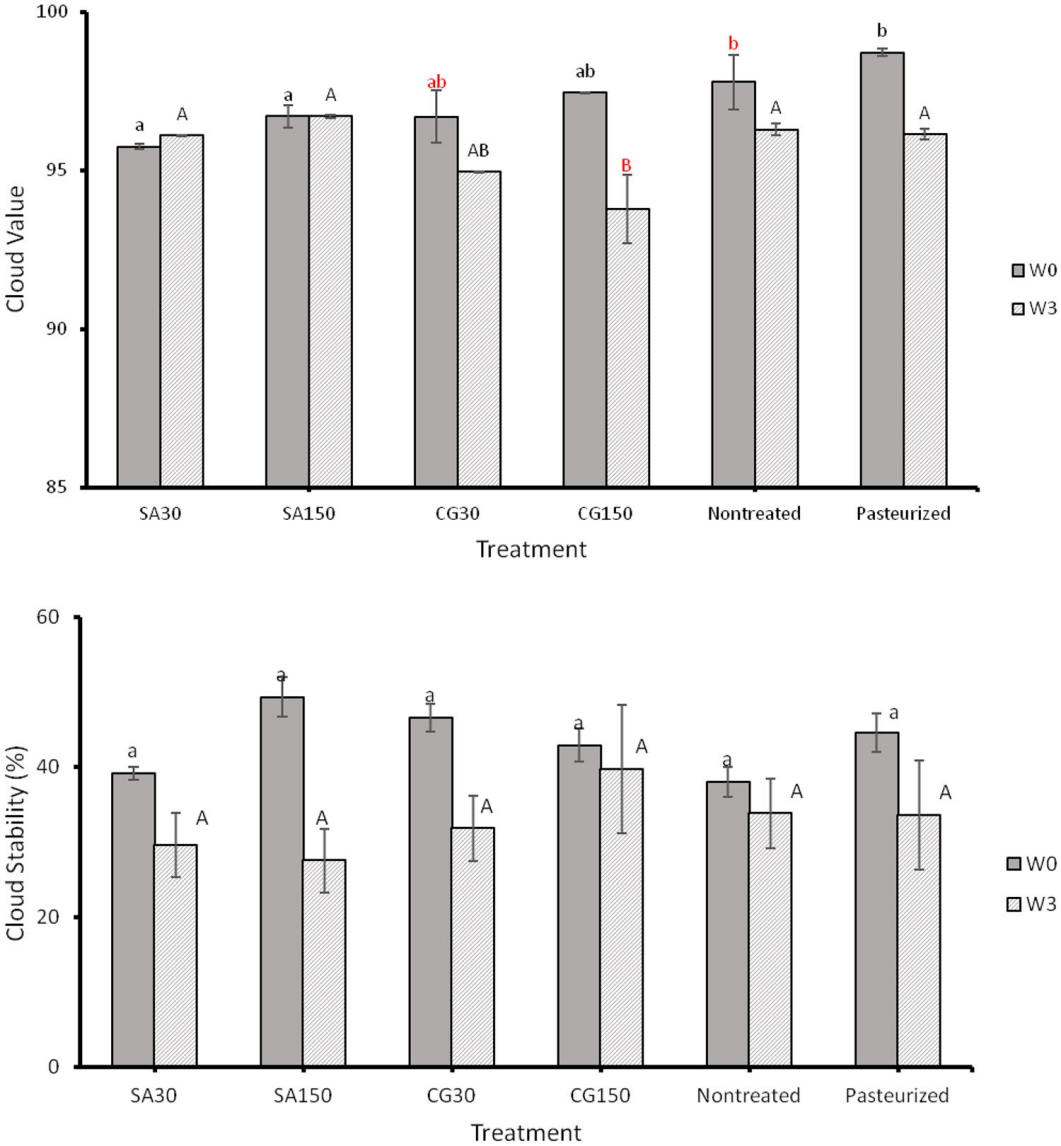
The effect of atmospheric cold plasma with simulated air (SA) (80% nitrogen and 20% oxygen) and combined gas (CG) (90% nitrogen and 10% oxygen) as working gasses for 30, 60, 90, 120, and 150 s processing times on cloud value and cloud stability of apple juice after the processing (W0) and after 3 weeks of storage at 4°C (W3). Values followed by different superscript letters are significantly different (*p* < 0.05).

Like other juices, cloud value and stability are critical factors in CAJ appearance and mouthfeel. Figure 2 and Table 3 show the effect of processing CAJ on cloud value, cloud stability, and PSD. After cold plasma processing with SA, the cloud values of CAJ were significantly lower compared to both pasteurized and untreated samples without storage. However, this difference was not observed when CG was used as a processing gas mixture (Figure 2). No changes were observed in the cloud value during the storage period. However, the cloud value of CG for 150 s and pasteurized CAJ decreased significantly. When comparing ACP‐treated samples, neither the treatment time nor the gas composition appeared to affect the cloud value. There were no significant differences in cloud stability among all samples before and after 3 weeks of storage compared to untreated and thermally pasteurized samples, and the influence of processing parameters on cloud stability was not statistically significant (Figure 2). Although the data are not visually represented in the accompanying figure, statistical analyses were performed to assess the effect of 3 weeks of storage on cloud stability within the same treatment conditions. The results revealed that cloud stability remained constant over time, except for the samples treated with SA for 150 s by ACP, which exhibited significantly lower values. Suspended pectin molecules mostly cause fruit juice cloud. Juice cloud loss is primarily caused by pectin deesterification by pectinesterase (PE). A free carboxyl group adjacent to a methyl ester group is attacked by PE, leading to methanol and polygalacturonic acid forming. Therefore, cloud loss is not desirable by consumers as they associate it with quality degradation and spoilage (Baker & Cameron, 1999; Krop & Pilnik, 1974).

**TABLE 3:** The effect of atmospheric cold plasma with simulated air (SA; 80% nitrogen and 20% oxygen) and combined gas (CG; 90% nitrogen and 10% oxygen) as feed gasses for 30, 60, 90, 120, and 150 s processing times on the particle size distribution (PSD) of apple juice after the processing (W0) and after 3 weeks of storage at 4°C (W3).

Storage	Treatment	*D*[4,3] (µm)	*D*[3,2] (µm)	*D*(0.1) (µm)	*D*(0.5) (µm)	*D*(0.9) (µm)	Span^*^
**0 days**	**SA30**	22.95 ± 3.36^ab^	2.56 ± 0.17^a^	1.11 ± 0.09 ^a^	8.43 ± 0.67 ^a^	48.32 ± 12.59 ^a^	5.56 ± 1.04 ^ab^
	**SA150**	23.25 ± 0.65 ^ab^	2.37 ± 0.41 ^a^	1.09 ± 0.21 ^a^	8.81 ± 0.53 ^a^	44.49 ± 0.42 ^a^	4.94 ± 0.27 ^ab^
	**CG30**	27.16 ± 2.00 ^b^	2.39 ± 0.26 ^a^	1.03 ± 0.12 ^a^	8.28 ± 0.21 ^a^	60.35 ± 7.11 ^a^	6.73 ± 0.42 ^b^
	**CG150**	18.65 ± 1.42 ^ab^	2.41 ± 0.07 ^a^	1.04 ± 0.06 ^a^	8.20 ± 0.38 ^a^	36.07 ± 2.09 ^a^	4.28 ± 0.46 ^a^
	**T0**	16.05 ± 1.97 ^ab^	2.69 ± 0.07 ^a^	1.23 ± 0.01 ^a^	7.91 ± 0.14 ^a^	34.04 ± 2.04 ^a^	4.14 ± 0.18 ^ab^
	**PAST**	19.05 ± 2.33 ^a^	2.37 ± 0.51 ^a^	0.87 ± 0.08 ^a^	8.93 ± 0.88 ^a^	41.85 ± 5.73 ^a^	4.63 ± 0.11 ^ab^
**3 weeks**	**SA30**	8.15 ± 0.94^A^	1.58 ± 0.45^A^	0.57 ± 0.12^A^	5.27 ± 0.13^A^	20.57 ± 3.39^A^	3.79 ± 0.58^ABC^
	**SA150**	20.13 ± 2.73^B^	2.91 ± 0.06^AB^	0.99 ± 0.02^A^	5.84 ± 0.16^A^	34.01 ± 5.02^A^	5.67 ± 1.01^C^
	**CG30**	10.38 ± 1.79^AB^	1.74 ± 0.48^A^	0.62 ± 0.10^A^	5.83 ± 0.08^A^	24.25 ± 1.73^A^	4.06 ± 0.23^AC^
	**CG150**	53.30 ± 3.85^C^	3.40 ± 0.81^B^	1.71 ± 0.45^B^	11.27 ± 0.37^B^	197.44 ± 24.60^B^	1.73 ± 0.16^B^
	**T0**	6.59 ± 0.86^A^	1.89 ± 0.09^A^	0.66 ± 0.02^A^	4.65 ± 0.21^A^	15.53 ± 3.11^A^	3.19 ± 0.52^AB^
	**PAST**	12.60 ± 5.61^AB^	1.55 ± 0.16^A^	0.54 ± 0.04^A^	5.86 ± 0.61^A^	27.54 ± 7.50^A^	4.57 ± 0.79^AC^

*Note*: Values followed by different superscript letters in the same column and in the same storage time are significantly different (*p* < 0.05).

* Span has no units.

The PSD of processed and non‐processed CAJ has demonstrated in Table 3. Overall, the particle volume mean diameter *D*[4,3] and the surface area mean diameter *D*[3,2] of ACP‐treated samples were not different from the untreated sample (*D*[4,3] =  16.05 ± 1.97 µm; *D*[3,2]  =  2.69 ± 0.07 µm) and the sample treated thermally (*D*[4,3]  =  19.05 ± 2.33 µm; *D*[3,2] = 2.37 ± 0.51 µm) except CG with 30 s before the storage. No significant effect of the gas type or processing time was observed on *D*[4,3] and *D*[3,2] values without storage. *D*[4,3] is significantly lowered when the plasma time for both gases was 30 s after the storage. Interestingly, there was a considerable increase in *D*[4,3] when the samples were treated with both gas mixtures for 150 s and stored over 3 weeks. After 3 weeks of storage, both *D*[4,3] and *D*[3,2] values showed a significant increase with longer treatment times, especially for samples treated with both plasma gases for 150 s. The terms *d*(0.1), *d*(0.5), and *d*(0.9) indicate that 10%, 50%, and 90% of the total particles are smaller than the measured size, respectively. *d*(0.1), *d*(0.5), and *d*(0.9) of the samples were not significantly different from one another, except that ACP treatment for CG showed significant increase (*p* < 0.05). The span value, the distribution width, of ACP‐treated samples ranged from 5.56 ± 1.04 to 4.94 ± 0.27 for SA and from 6.73 ± 0.42 to 4.28 ± 0.46 for CG, which are not significant (*p* < 0.05) different from non‐processed and thermally processed samples. After 3 weeks of shelf life, the span of ACP samples with CG with 150 s dropped significantly. The span value indicated the homogenization of the samples, and the samples with a lower span value demonstrated more cloud stability (Illera et al., 2019).

### 3.3 Effect of ACP on antioxidant activity and phenolic content of apple juice

**FIGURE 3:**
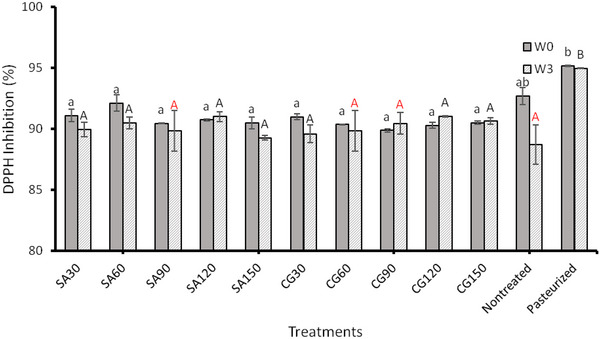
The effect of atmospheric cold plasma with simulated air (SA) (80% nitrogen and 20% oxygen) and combined gas (CG) (90% nitrogen and 10% oxygen) as working gases for 30, 60, 90, 120, and 150 s processing times on antioxidant activity in apple juice after the processing (W0) and after 3 weeks of storage at 4°C (W3). Values followed by different superscript letters are significantly different (*p* < 0.05).

An antioxidant scavenging assay using the antioxidant‐reducing capacity against the DPPH free radical was conducted to compare the antioxidant capacities of processed and unprocessed CAJ (Figure 3). As a result of the ACP processing, antioxidant activity did not significantly change as compared to unprocessed juices. Dasan and Boyaci (2018) found a slight decrease in antioxidant capacity and postulated that free radicals generated by ACP may react with antioxidant compounds in apple juice and decrease their concentration. A significant increase in DPPH inhibition was observed in thermally pasteurized juices (*p* < 0.05). The results had a similar trend after 3 weeks of storage. Neither ACP‐treated nor thermally pasteurized juices were affected by the storage, whereas raw juices lost significant antioxidant capacity. The impact of gas mixtures and processing times on the antioxidant activities of ACP‐treated CAJ was found to be insignificant. Liao et al. (2018) and Almeida et al. (2015) treated apple and prebiotic orange juices, respectively. It was found that plasma treatment of both juices reduced their antioxidant capacity slightly but not significantly. According to Wang et al. (2023), plasma treatment times more than 2 min significantly decreased antioxidant activity. The results were similar to those of Pankaj et al. (2017) and Rodríguez et al. (2017), which used grape juice and cashew apple juice, respectively. Nevertheless, this study used a much greater amount of 100 mL juice than used in previous studies. Similarly to most other quality parameters, the significance of the ACP processing in antioxidant capacity varies with juice amount, treatment time, and so forth.

**FIGURE 4:**
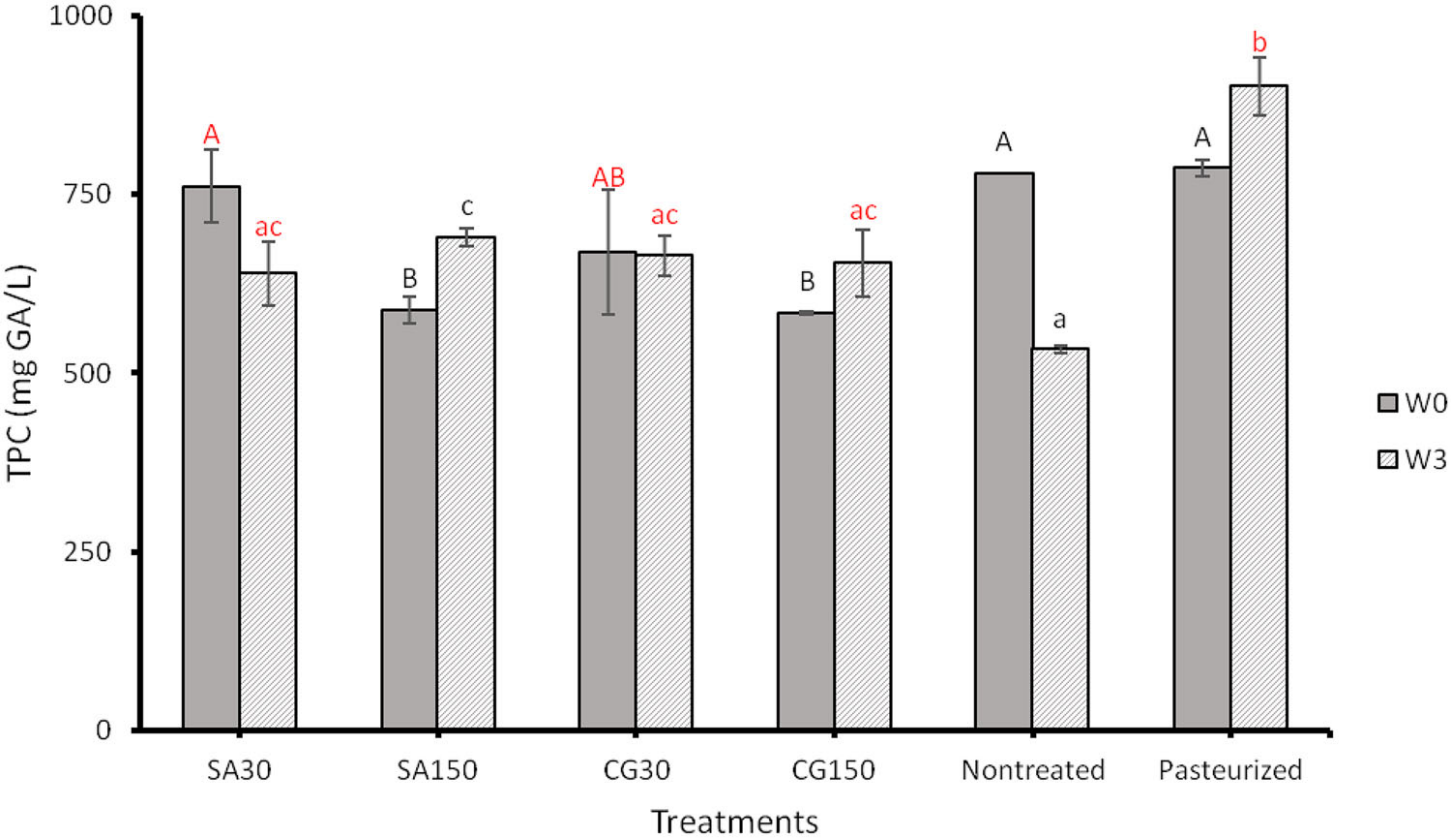
The effect of atmospheric cold plasma with simulated air (SA) (80% nitrogen and 20% oxygen) and combined gas (CG) (90% nitrogen and 10% oxygen) as working gases for 30, 60, 90, 120, and 150 s processing times on total phenolic content (TPC) in apple juice after the processing (W0) and after 3 weeks of storage at 4°C (W3). Values followed by different superscript letters are significantly different (*p* < 0.05).

The TPC of ACP‐treated samples significantly decreased by increasing the plasma treatment time compared to unprocessed and thermally processed samples (Figure 4). There was no significant influence of plasma gas type on the phenolic content. However, after 3 weeks of storage, the phenolic content for samples with shorter plasma treatment times decreased, and for longer plasma treatment times, it increased. It should be noted that after the storage, the phenolic content of plasma‐treated samples was higher than that of unprocessed juices and lower than the thermally processed ones. Several studies have observed a decrease in TPC of juices following the APC treatment (Almeida et al., 2015; Liao et al., 2018; Pankaj et al., 2017). Conversely, other studies have reported the increased value of juices’ TPC after treatment with cold plasma (Bursac Kovacevic et al., 2016; Garofulic et al., 2015; Herceg et al., 2016). The reduction in TPC is thought to be due to their susceptibility to ozone, which is produced during cold plasma treatment. Ozone has been shown to degrade the aromatic rings of phenolic compounds effectively (Perez et al., 2002; Stalter et al., 2011). On the other hand, the increase in TPC could be linked to the disruption of plant cell walls, allowing the release of phenolic compounds into the surrounding medium, resulting in higher concentrations (Landbo & Meyer, 2001).

Ozen et al. (2022) used OAS to examine the composition of SA and CG plasma. The absorbance spectrum, ranging from 190 to 308 nm, revealed the presence of ozone (200–300 nm). Given that CAJ was used in this study, it can be inferred that the plasma‐assisted breakdown of cell walls during treatment contributed to increased phenolic content with increase in the treatment duration. A similar trend was observed in changes in antioxidant activity (Figure 3), which is consistent with the reported correlation between TPC and antioxidant activity in foods (Muhammad et al., 2018).

### 3.4 Effect of ACP on microbial reduction

Pasteurized CAJs were inoculated with *A. acidoterrestris* spores and then treated with ACP for 3 min with SA and CG feed gas mixtures. Although ACP proved effective for killing microorganisms in fruit juices (Ozen & Singh, 2020), it proved less effective for killing spores. The results showed 1.06 ± 0.35 log CFU/mL reduction after 3 min of processing with SA and 0.15 ± 0.07 log CFU/mL reduction after 3 min processing with CG. Unlike vegetative cells, bacterial spores have hard and multilayered coats, making them more difficult to inactivate by ACP (Tseng et al., 2012). Wang et al. (2022) observed 4.9 log CFU/mL reduction in vegetative cell counts of *A. acidoterrestris* in 2 min in 0.85% sterile saline solution with ACP generated by 30 kV power. No other study has been conducted on the inactivation of *A. acidoterrestris* spores.

**FIGURE 5:**
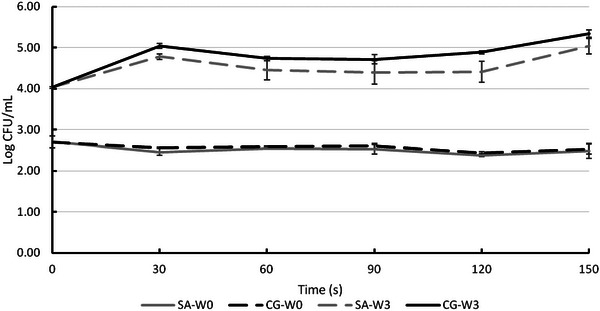
The effect of atmospheric cold plasma with simulated air (SA; 80% nitrogen and 20% oxygen) and combined gas (CG; 90% nitrogen and 10% oxygen) as feed gases for 30, 60, 90, 120, and 150 s processing times on total yeast and mold counts of apple juice just after the processing (W0) and 3 weeks shelf life (W3) at 4°C. The sample groups are represented as follows: SA‐W0: simulated air‐treated juice, immediately analyzed; CG‐W0: combined gas‐treated juice, immediately analyzed; SA‐W3: simulated air‐treated juice, analyzed after 3 weeks of storage; CG‐W3: combined gas‐treated juice, analyzed after 3 weeks of storage.

No bacterial growth was detected in any of the samples, even after 3 weeks of storage, as the acidic environment of apple juice (pH 3.8) was unsuitable for bacterial survival. The effects of ACP processing on yeast and mold (YMC) survival are presented in Figure 5. The unprocessed apple juice contained 2.70 ± 0.14 log CFU/mL of YMC, and ACP treatment had no significant effect on YMC. All samples except thermally pasteurized juices showed a significant increase in YMC counts over 3 weeks of storage. ACP was used in processing 5 mL tomato juice by Starek et al. (2019), which resulted in inactivation of all yeasts and molds after 5 min of processing. In this study, 100 mL samples and shorter processing times were used; thus, YMC inactivation were expected to be lower or not at all, as YMC have chitin‐containing cell walls, making them more resistant than bacteria (Liao et al., 2017).

## 4 CONCLUSIONS

This study strongly suggests that ACP is an effective nonthermal method for preserving CAJ, offering several advantages over conventional thermal pasteurization. ACP treatment preserved critical physicochemical properties, such as pH, acidity, °Brix, and viscosity, with no immediate changes observed after treatment. ACP‐treated samples showed remarkable stability in color and cloud stability, which are important for both visual appeal and texture. Even after 3 weeks of storage, ACP prevented darkening and cloud loss, outperforming thermal methods that caused significant degradation in these properties. One of the standout features of ACP is its ability to maintain and even enhance the nutritional value of CAJ compared untreated juices. ACP preserved antioxidant activity, and TPC, with a notable increase in TPC, was observed after storage. This enhancement was likely due to plasma‐induced cell wall disruption, which highlights ACP's potential to improve bioactive compound availability, making it a valuable option for functional food applications. From a microbiological perspective, ACP demonstrated limited efficacy in reducing bacterial spores. However, this finding highlights the need for further research to optimize plasma treatment conditions for spore inactivation. Future studies should explore factors such as varying plasma parameters (e.g., treatment time, gas composition, and power settings), as well as their effects on different types of spores, to improve the efficacy of ACP in achieving reliable spore inactivation. Additionally, combining ACP with other antimicrobial strategies could offer promising results for more effective spore reduction. By combining microbial safety with superior preservation of sensory, physical, and nutritional quality, ACP stands out as a sustainable, consumer‐friendly alternative to thermal pasteurization. Its scalability and energy efficiency make it an attractive option for the juice industry. Future research should include optimization of treatment variables and explore hurdle approach to improve spore inactivation while retaining ACP's unique benefits. This study highlights ACP as a key technology for the next generation of food preservation, balancing safety, quality, and sustainability.

## AUTHOR CONTRIBUTIONS

**Emine Ozen**: Conceptualization; data curation; formal analysis; investigation; methodology; writing—original draft. **Abhinav Mishra**: Resources; validation; software. **Rakesh K. Singh**: Conceptualization; supervision; funding acquisition; project administration; resources; writing—review and editing; investigation.

## CONFLICT OF INTEREST STATEMENT

Authors confirm that there are no financial interests or personal connections that might potentially bias or influence the findings presented in this study.

## FUNDING INFORMATION

This study was conducted without specific financial support from governmental, commercial, or non‐profit organizations.
